# Quantitative MR Imaging Marker: Effective Cross-Sectional Area of the rotator cuff and Its Correlation with the Biodex Isokinetic Test

**DOI:** 10.2174/0115734056402394250921003713

**Published:** 2025-10-02

**Authors:** Kyu-Chong Lee, Woong Kyo Jeong, Kyung-Sik Ahn, Woo Young Kang, Baek Hyun Kim, Euddeum Shim, Hee-Gone Lee, Yeo Eun Han, Chang Ho Kang

**Affiliations:** 1 Department of Radiology, Korea University Anam Hospital, Seoul, Republic of Korea; 2 Department of Orthopedics, Korea University Anam Hospital, Seoul, Republic of Korea; 3 Department of Radiology, Korea University Guro Hospital, Seoul, Republic of Korea; 4 Department of Radiology, Korea University Ansan Hospital, Ansan, Republic of Korea

**Keywords:** rotator cuff muscle, Effective cross-sectional area, Isokinetic test, Dixon MRI, Fat fraction, Peak torque

## Abstract

**Objective:**

To evaluate the correlation between the effective cross-sectional area (eCSA) of the rotator cuff muscle measured using Dixon MRI and the outcomes of the Biodex Isokinetic Test.

**Methods:**

The cross-sectional area (CSA) of the subscapularis (SSc), supraspinatus (SST), and infraspinatus+teres minor (ISTM) muscles of 87 patients who had undergone shoulder MRI and Biodex Isokinetic Test were measured in the oblique sagittal Y-view. The eCSA was calculated by multiplying the CSA by (1-fat fraction). Eight shoulder movements (FL60, EX60, FL180, EX180, ER60, IR60, ER180, and IR180) each assessed using four parameters (peak torque [PT], peak torque/body weight, torque at 30° [TQ30], and total work) were recorded on Biodex. Pearson correlation coefficients were calculated between eCSA and Biodex outcomes. Univariate regression analyses were conducted to identify the factors influencing the Biodex results. General linear models were applied to confirm the correlations between the eCSA and 32 Biodex parameters after adjusting for these factors.

**Results:**

The eCSA of the SSc, SST, and ISTM exhibited significant correlations with TQ30 at IR180 (r=0.549) and FL60 (r=0.522), PT at ER60 (r=0.656) and EX60 (r=0.575), and PT at ER60 (r=0.674) and FL180 (r=0.626), respectively. Age, sex, SST, and SSc tears were identified as factors influencing the Biodex results. FL60TQ30, EX60PT, and ER60PT exhibited significant associations with the eCSA of SSc, SST, and ISTM, respectively, after adjusting for these factors.

**Conclusion:**

eCSA may be a useful quantitative imaging marker for assessing the function of the rotator cuff muscle. FL60TQ30, EX60PT, and ER60PT are useful Biodex indices for SSc, SST, and ISTM, respectively.

## 1 INTRODUCTION

The Biodex Isokinetic Test is used to evaluate motor function based on the complex interactions between various muscles. It is typically used to assess the function of major joints, such as the hip, knee, and shoulder. This test can objectively measure muscle performance, facilitating decision-making related to diagnosis, treatment, and rehabilitation. The results of the Biodex Isokinetic Test include various factors such as the peak torque (PT), peak torque/body weight (BW), torque at 30° (TQ30), and total work (TW). Each of these values is associated with specific exercises such as extension (EX), flexion (FL), internal rotation (IR), and external rotation (ER) of each joint. The interpretation of the test outcomes can be challenging owing to the multitude of result values, with some results exhibiting low levels of reliability [1]. Also, the results of the Biodex Isokinetic Test do not always correlate with those of physical examination [2]. Furthermore, given the intricate effects of various muscles related to single movement, the use of this test to interpret the influence of individual muscle is limited. The rotator cuff plays a crucial role in maintaining the stability and movement of the shoulder joint. It generates various movements by collaborating with the pectoralis major/minor muscles and deltoid muscles, thereby influencing the results of the Biodex Isokinetic Test [3, 4]. However, the Biodex outcomes that are specifically affected by the rotator cuff remain unclear, and conducting this test is not always feasible in clinical settings [4]. The mass of the rotator cuff muscle and the degree of fat degeneration, which are associated with the severity of rotator cuff tears, can predict functional outcomes after repair [5, 6]. The Goutallier classification, a non-quantitative visual grading system, is used to assess the extent of fat degeneration in the rotator cuff muscles [7]; however, it has limitations in terms of reproducibility and sensitivity. Although this classification system is easy and quick assessment tool, the reproducibility is relatively low. Moreover, it cannot effectively discriminate subtle differences, particularly during the follow-up. A Dixon-based water–fat separation technique, developed to quantitatively assess the fat fraction in the rotator cuff muscles to overcome these limitations [6], exhibits high reliability even in muscles with low-fat content [8]. Muscle movement is generated by the contractile muscle portion, excluding fat infiltration; therefore, a novel quantitative imaging marker called the “effective cross-sectional area (eCSA),” calculated by multiplying the cross-sectional area (CSA) of the rotator cuff muscle in the Y-view by the (1-fat fraction) measured in the Dixon sequence, was introduced. To the best of our knowledge, no previous study has explored the relationship between the eCSA of the rotator cuff muscle and the outcomes of the Biodex Isokinetic Test. Therefore, this study aimed to analyze the correlation between the new imaging marker named eCSA of the rotator cuff muscle and the outcomes of the Biodex Isokinetic Test to determine the feasibility of using eCSA as an imaging marker and identify key Biodex parameters related to the specific function of the rotator cuff muscles.

## 2 MATERIALS AND METHODS

### 2.1 Ethics Statement

This study was approved by the Institutional Review Board and Ethics Committee of Korea University Anam Hospital (IRB no.2023AN0559). The requirement for obtaining informed consent from the participants was waived as the data were collected retrospectively and analyzed anonymously. This study complied with the ethical principles of the Declaration of Helsinki of 1964, as revised by the World Medical Association in Edinburgh in 2000.

### 2.2 Study Population

Patients who had undergone rotator cuff repair or reverse total shoulder arthroplasty (RTSA) between January 2017 and December 2021 with the results of both the shoulder MRI and Biodex Isokinetic Test were eligible for inclusion in this study. Among the 166 eligible patients, those with missing values for any of the 32 Biodex Isokinetic Test results (n=48), those who did not undergo MRI before the surgery, those with an interval of >6 months between the Biodex Isokinetic Test and MRI (n=30), and those with poor MRI quality that hindered evaluation (n=1) were excluded. Finally, 87 participants were included in the analysis, of which the right and left shoulders of 58 and 26 patients were affected, respectively.

### 2.3 Image Acquisition

MRI was performed using the following 3-Tesla machines: Skyra (n=34), Prisma (n=36) (Siemens, Erlangen, Germany), and Achieva (n=17) (Philips, Best, The Netherlands). All MR scans were conducted with the patients lying in the supine position, with their shoulder joints positioned neutrally and their palms facing upwards. All imaging planes were perpendicular to the glenohumeral joint. The standard protocol comprised the acquisition of T1-weighted oblique coronal and sagittal images, proton-weighted axial images with fat saturation, and T2-weighted oblique coronal and sagittal images obtained using the water-fat separation technique (Dixon) with a dedicated shoulder coil. The field of view and slice thickness were set as 160×160 mm and 2.5–3.0 mm without a gap for all sequences, respectively.

### 2.4 Effective Cross-sectional Area Measurement

**Fig. (1):**
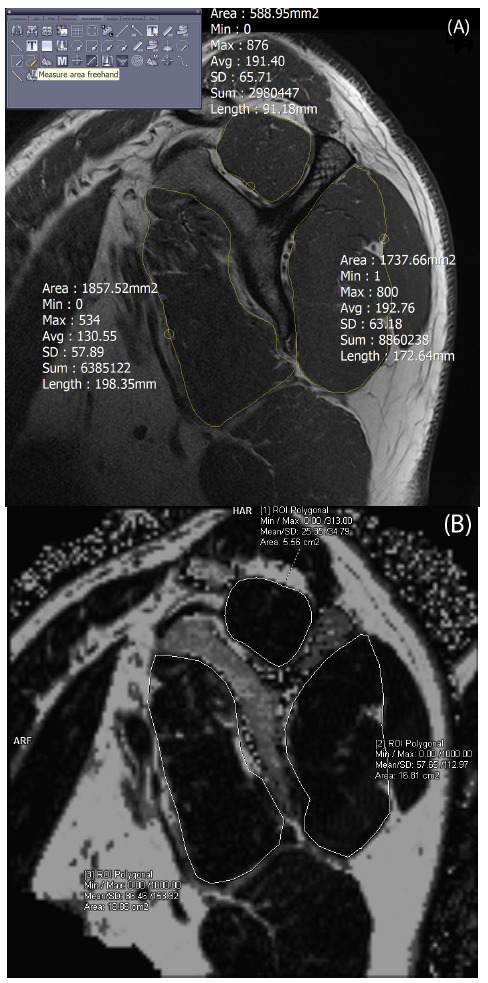
Measurement of the effective cross-sectional area (CSA) of the three muscle groups. (**A**) The CSA was measured using a freehand measuring tool on Infinitt PACS. The outer margin of the three muscle groups (subscapularis, supraspinatus, infraspinatus+teres minor) was drawn on the sagittal Y-view. (**B**) Fat fraction was automatically calculated on the Dixon MRI sequences using Syngo.via.

The CSA was measured using a freehand measuring tool on an Infinitt PACS Viewer (Infinitt Healthcare, Korea). A musculoskeletal radiologist with 4 years of clinical experience drew the outer margin of each of the three muscle groups in a sagittal Y-view as follows: subscapularis (SSc), supraspinatus (SST), and infraspinatus plus teres minor (ISTM) (Fig. **1a**). The radiologist was blinded to patient information such as age, sex, and tendon tears. The fat fraction was calculated by Syngo.via (Siemens Healthineers, Forchheim, Germany) based on the Dixon sequence (Fig. **1b**). The eCSA was calculated by multiplying the CSA by (1-fat fraction).

### 2.5 Clinical Data Acquisition

Data regarding age, sex, body mass index (BMI), and history of hypertension and diabetes mellitus were obtained by reviewing the electronic medical charts of the patients. In addition, data regarding lifestyle habits, such as smoking status and alcohol consumption, were obtained. Tears of the SST, IST, and SSc tendons, as well as that of the long head of the biceps tendon (LHBT), were documented based on surgical records. The SST and IST tendon tears were categorized as normal, partial-thickness, or full-thickness tears. LHBT tears were classified into three categories: normal, partial tear, and total rupture. The Yoo and Rhee classification was used to classify the SSc tendon tears [9]. Fraying or a longitudinal split tear of the leading edge, which corresponds to the cranial part of the SSc tendon, was defined as a Yoo and Rhee type I tear. Type IIA tear is characterized by <50% detachment of the SSc tendon at the first facet, whereas type IIB is characterized by >50% detachment of the SSc tendon at the first facet, corresponding to approximately one-quarter to one-third of the entire SSc tendon length. Type III tear is characterized by the involvement of the entire SSc tendon at the first facet, including the lateral hood. Type IV tear is characterized by the exposure of the first and second facets. Type V is defined as a complete tear involving the muscular portion [9, 10].

### 2.6 Biodex Isokinetic Test

**Fig. (2):**
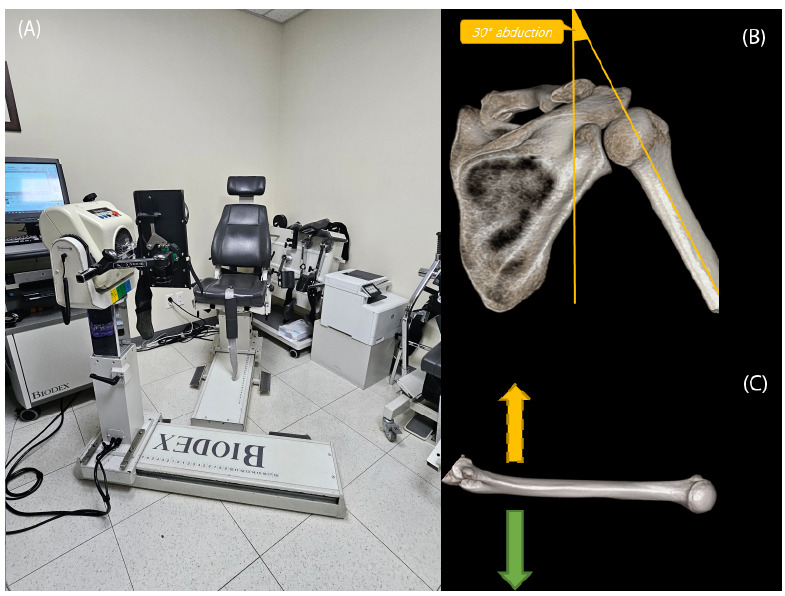
(**A**) Biodex dynamometer (Biodex System 4 Pro) installed in our hospital. (**B**) Schematic image of flexion (FL) and extension (EX) of the shoulder with an angular velocity of 60°/s and 180°/s. The arm was abducted by approximately 30°. Upward and downward movement of the arm resulted in flexion and extension of the shoulder. (**C**) Schematic image of external rotation (ER, arrow) and internal rotation (IR, dashed arrow) of the shoulder with an angular velocity of 60°/s and 180°/s. All 32 Biodex parameters (eight movements with each of the four parameters) were measured automatically and scored using this dynamometer.

An Isokinetic shoulder strength test was performed using a Biodex dynamometer (Biodex System 4 Pro; Biodex Medical Systems, Shirley, NY, USA) (Fig. **2a**). All results were automatically measured and recorded using a dynamometer. The length of the chain and degree of joint flexion were adjusted to assess different muscle groups. Each patient was seated comfortably on the Biodex chair with their trunk upright, and the hips and knees were flexed at an angle of approximately 85°. The arm was abducted by approximately 30° while seated to align with the horizontal axis of the glenohumeral joint. Upward and downward movement of the arm made FL and EX (Fig. **2b**), whereas ER and IR were defined as the movement rotating the arm in a modified neutral position (Fig. **2c**). The FL, EX, ER, and IR movements performed at two different angular velocities (60°/s and 180°/s) yielded the following eight shoulder movements: FL60, EX60, FL180, EX180, ER60, IR60, ER180, and IR180. Four parameters, PT, BW, TQ30, and TW, were measured for each of these movements. Thus, the following 32 parameters were measured for each patient: PT of FL60, BW of FL60, TQ30 of FL60, TW of FL60, PT of EX60, BW of EX60, TQ30 of EX60, TW of EX60, PT of FL180, BW of FL180, TQ30 of FL180, TW of FL180, PT of EX180, BW of EX180, TQ30 of EX180, TW of EX180, PT of ER60, BW of ER60, TQ30 of ER60, TW of ER60, PT of IR60, BW of IR60, TQ30 of IR60, TW of IR60, PT of ER180, BW of ER180, TQ30 of ER180, TW of ER180, PT of IR180, BW of IR180, TQ30 of IR180, and TW of IR180.

### 2.7 Statistical Analysis

Patient characteristics were compared according to sex. Continuous variables were compared using independent-sample t-tests, whereas categorical variables were compared using Pearson’s chi-squared or Fisher’s exact test. Pearson correlation coefficients between the eCSA of the rotator cuff muscle and 32 Biodex Isokinetic Test parameters were calculated to determine whether the novel quantitative imaging marker, the eCSA, was associated with the outcomes of the Biodex Isokinetic Test. The Pearson correlation coefficients and *p*-values were calculated, and the results were presented in a correlation coefficient matrix heatmap. Higher values are indicated with darker shading. The correlation coefficients were interpreted as follows: <0.2, poor; 0.21–0.5, fair; 0.51–0.8, moderate; and 0.81–<1.0, strong positive correlation [11]. Univariate regression analyses were conducted for all 32 Biodex Isokinetic Test parameters using the clinical data of all patients, including sex, age, BMI, rotator cuff tear status, underlying diseases, and lifestyle habits that could have influenced the outcomes of the Biodex Isokinetic Test. The variables that exhibited significant associations with the outcomes of the Biodex Isokinetic Test with *p*-values of <0.05 in the univariate regression analyses were adjusted for in the final general linear model (GLM) analyses to assess the relationship between the eCSA of the SSc, SST, and ISTM and the 32 Biodex Isokinetic Test parameters. The Akaike Information Criterion (AIC) was analyzed for each GLM to assess model fit. All statistical analyses were conducted using R version 4.4.1 (R Foundation for Statistical Computing, Vienna, Austria). Statistical significance was set at p <0.05.

## 3 RESULTS

### 3.1 Patient Characteristics

Eighty-seven participants, including 45 females and 42 males, were enrolled in this study. Table **1** presents the baseline characteristics of the patients according to sex. All 32 parameters of the Biodex Isokinetic Test results differed significantly between the male and female patients. Furthermore, the eCSA of the SST, SSc, and ISTM differed significantly between the male and female participants. However, no significant differences were observed between the male and female participants in terms of BMI, medical history, and presence of rotator cuff or LHBT tears.

### 3.2 Correlation between the eCSA and Biodex Parameters

Fig. (**3**) presents the correlation matrix heatmap, displaying the Pearson correlation coefficients between the three eCSAs and all 32 Biodex parameters. Comparison between the four key parameters (PT, BW, TQ30, and TW) for each movement revealed that BW generally exhibited a lower degree of correlation compared to that by the other three parameters. IR180TQ30 exhibited the highest correlation coefficient for the eCSA of the SSc (0.549), followed by FL60TQ30 (0.53). This finding indicates the presence of moderate positive correlations. ER60PT exhibited the highest correlation coefficient for the eCSA of SST and ISTM, with values of 0.656 for SST and 0.674 for IST, indicating moderate positive correlations. Excluding the ER movement (both ER60 and ER180), SST exhibited the next highest correlation with EX60PT (0.575), whereas ISTM exhibited a higher value with FL180PT (0.626).

A relatively high correlation coefficient was observed with internal rotation for SSc, whereas a higher correlation was observed with external rotation for ISTM. SST exhibited a high correlation with external rotation, but it was lower than that of ISTM.

### 3.3 Final GLMs Adjusted for Multiple Variables Exhibiting the Association between eCSA and the Biodex Outcomes

#### 3.3.1 eCSA of SSC

Table **2** presents the results of the univariate regression analyses for the key Biodex parameters that exhibited the highest correlation with the eCSA of SSc in the previous correlation matrix, as well as the GLM model that includes the eCSA of SSc. The results of the final GLM revealed that the eCSA of the SSc was not a significant variable for IR180/TQ30. However, eCSA of SSc remained a significant factor for FL60/TQ30, even after adjusting for all other variables.

#### 3.3.2 eCSA of SST

Table **3** presents the results of the univariate regression analyses for the key Biodex parameters exhibiting the highest correlation with the eCSA of SST in the previous correlation matrix, as well as the GLM model that includes the eCSA of SST. The final GLM analysis for ER60PT, which exhibited the highest correlation coefficient, revealed that the eCSA of SST remained significant even after adjusting for all other variables. In addition, age and sex were identified as significant factors in the final GLM. Increasing age was associated with a decrease in ER60PT. Furthermore, the ER60PT values of the male participants were higher than those of the female participants. Notably, a trend toward an increase in the ER60PT values was observed when the SSc tear extended beyond the second facet. The eCSA of SST remained significant in the final GLM of EX60PT after adjusting for all other variables. The EX60PT values decreased with increasing age, current smoking status, and the presence of partial tears in the SST or SSc.

#### 3.3.3 eCSA of ISTM

Table **4** presents the results of the univariate regression analyses of the key Biodex parameters that exhibited the highest correlation with the eCSA of ISTM in the previous correlation matrix, along with the GLM model that includes the eCSA of ISTM. The final GLM analysis for ER60PT, which exhibited the highest correlation coefficient, revealed that the eCSA of the ISTM remained significant even after adjusting for all other variables. Similar to the results of the final GLM for EX60PT with the eCSA of SST, age, sex, and SSc tears were identified as significant factors in the final GLM. The final GLM for FL180PT revealed that the eCSA of ISTM remained significant after adjusting for all other variables and that male sex exhibited a significant association with increased FL180PT values.

#### 3.3.4 Comparison of AIC

As the eCSA of SST and ISTM exhibited significant correlations with ER60PT, the AIC values of both models were compared. The AICs were 463.43 and 461.74 for the eCSA of the SST and ISTM, respectively. As a lower AIC value indicates a more suitable model, the eCSA of ISTM was presumed a more suitable model.

## 4 DISCUSSION

The present study demonstrated that the eCSA of the rotator cuff muscles exhibits statistically significant correlations with multiple parameters of the Biodex Isokinetic Test. Furthermore, these correlations persisted even after adjusting for other compounding variables that may affect the results of the Biodex Isokinetic Test, such as age and sex. These findings indicate that the eCSA of the rotator cuff muscles can be used as a reliable and objective quantitative imaging marker for assessing the function of the rotator cuff muscle. The eCSA of the rotator cuff muscles, extracted from pre-operative MRI, may provide functional information regarding the shoulder function in patients who face difficulty in performing the Biodex Isokinetic Test.

The Pearson correlation matrix between the eCSA and Biodex parameters revealed that the eCSA of SSc exhibited a strong correlation with internal rotation (IR60 and IR180). In contrast, the eCSA of ISTM exhibited a high correlation with external rotation (ER60 and ER180). Given that SSc and ISTM play a significant role in internal and external rotations, respectively, these findings suggest that the eCSA is closely associated with muscle function. The eCSA values of SSc and ISTM were similar across sexes (Table **1**). This finding is consistent with that of previous studies, which reported no significant differences in muscle volume between SSc and ISTM in non-pathological shoulders based on the computed tomography (CT) findings [12]. The opposing anatomical positions and functional roles of these muscles highlight the importance of the anterior (SSc) to posterior (IST+TM) rotator cuff transverse force coupling [12, 13]. SST primarily plays a role in abduction; However, in our hospital, the Biodex system did not include a specific abduction movement assessment. Nevertheless, the eCSA of SST exhibited moderate correlations across most movements, as SST is generally important for all shoulder movements.

The CSA in various regions was associated with muscle strength and function, as measured using a dynamometer, in previous studies [14-19]. Ultrasonography, CT, and MRI have been used to measure the CSA of muscles. Among these imaging modalities, ultrasonography yields high reproducibility and accuracy [14, 15]. Forbush *et al.* reported an association between the CSA of SST, as measured using ultrasonography during diagonal horizontal adduction, and the results of the dynamometer test [14], indicating that the CSA of the shoulder muscle could be a useful imaging marker for muscle function. Previous studies have assessed the CSA measured using MRI in various regions [17-19]. Lee *et al.* revealed that the CSA of the back muscles and the CSA ratio of the back muscles to discs exhibited significant associations with the results of the Biodex Isokinetic Test in patients with back pain [17]. Aisha *et al.* compared the CSA and fat fraction of the back and lower limb muscles using the Dixon method and Biodex Isokinetic Test in patients with Becker muscular dystrophy and revealed that compared with the Biodex Isokinetic Test, quantitative MRI facilitated more sensitive detection of changes [18]. Oh *et al.* reported a link between the CSA of the rotator cuff muscle, as measured using MRI, and the prognosis of patients undergoing rotator cuff repair [19]. In addition, Rouleau *et al.* reported that degeneration of the rotator cuff muscles measured by ultrasound had a negative impact on the prognosis of proximal humerus, greater tuberosity fracture [20]. The application of a novel quantitative imaging marker, the eCSA, to the rotator cuff muscle in the present study revealed its association with the results of the Biodex Isokinetic Test. This finding demonstrates its potential as an imaging marker for evaluating muscle function.

The eCSA is the value obtained by excluding the fat fraction from the simple CSA. As intramuscular fat increases, the mechanical properties of muscle fibers change, leading to impaired muscle contraction and reduced muscle power [21-23]. Therefore, the area excluding these fatty degenerative portions can be considered the substantial part that reflects actual muscle function. Regarding muscle degeneration, the widely used Goutallier grade [7], as a categorical variable, is limited in its ability to capture subtle changes in muscle quality. In contrast, eCSA, which reflects both muscle quantity and quality, provides a continuous and quantitative measure that we believe is a more meaningful indicator of muscle status. In our study cohort, only two patients who underwent RTSA exhibited a Goutallier grade of IV, while the majority of patients had grades of II or lower, demonstrating minimal variation. These findings suggest that eCSA, as a quantitative imaging marker, may offer a more precise reflection of muscle function compared to conventional categorical assessments.

The Pearson correlation heatmap revealed that PT exhibited relatively high correlation values with the following muscle groups: SSc, SST, and ISTM. Similarly, TQ30 and TW also exhibited some darker shaded areas in the heatmap, indicating the presence of relatively high and meaningful correlations. However, BW, which represents PT corrected for body weight, did not exhibit a meaningful association with eCSA. This may be attributed to the eCSA not being adjusted for the body weight of the patient in the present study. Meanwhile, PT, which represents maximal muscle strength, was generally higher at 60°/s in our study. This is because lower angular velocity (60°/s) primarily evaluates maximal muscle strength under slower, more controlled movements, whereas higher angular velocity (180°/s) emphasizes muscle power and endurance by simulating faster, more functional activities [24]. Although TQ30 and TW partially reflect muscular endurance, their values can vary depending on participant characteristics and fatigue levels; therefore, in our study, higher values were not consistently observed at 180°/s.

The final GLM for SSc exhibited a significant effect on FL60TQ30, which had the second-highest correlation coefficient, even after adjusting for other variables affecting the results of the Biodex test. SST and ISTM demonstrated the strongest correlation with ER60PT. Notably, SST and ISTM continued to exhibit a significant effect even after adjusting for other variables. The AIC values indicated that the eCSA of ISTM achieved a better fit with ER60PT than the eCSA of SST. SST is a major shoulder abductor; however, it also plays a significant role in facilitating external rotation alongside ISTM, which is consistent with the findings of the present study [25-27]. However, the GLM model for ER60PT was considered to exhibit better results with the eCSA of ISTM than with the eCSA of SST, based on the AIC values, since ISTM plays a more primary role in external rotation. SST exhibited a significant effect on EX60PT, which exhibited the next highest correlation coefficient even after adjustments. Therefore, EX60PT, ER60PT, and FL60TQ30 are important Biodex indices for assessing the function of SST, ISTM, and SSc, respectively.

Age, sex, and SST and SSc tears significantly influenced the results of the Biodex test in the final GLM models. Younger age and male sex were associated with higher Biodex values. This finding is consistent with those of previous studies indicating that the muscle strength in men is generally greater than that in women and that muscle function and strength decrease with age [28-32]. In the case of patients with SST tendon tears, the findings presented in Table 3 indicate that all results tended to negatively impact the outcomes of the Biodex test in the univariate regression analysis. Furthermore, partial tendons in the SST tendon exhibited a significant association with lower Biodex values in the final GLM model of the eCSA of SST at EX60PT. These findings are consistent with those of previous studies [25, 33], which demonstrated that SST tendon tears adversely affect Biodex outcomes. Furthermore, the effect of the eCSA of SST and SST tendon tears on the final GLM for EX60PT emphasized the importance of the EX60PT index in assessing SST function. Tears in the SSc tendon exhibited a significant difference in the univariate regression for ER60PT and in the final GLM model for the eCSA of SST and ISTM at ER60PT. Notably, the Biodex values were significantly increased in cases with Yoo and Rhee grade IIb or higher tears. Although SSc functions as a major internal rotator, the IR is often preserved in patients with tears in the SSc tendon owing to the other internal rotator muscles compensating for this [34]. Consistent with this finding, the SSc tendon tears did not exhibit statistically significant differences in terms of IR movement in the present study. However, the increase in ER60PT values in cases of high-grade SSc tendon tears (Yoo and Rhee IIb or higher) may be attributed to an imbalance in the previously mentioned rotator cuff transverse force coupling [12, 13]. This imbalance between SSc and ISTM probably results in the relatively high ER60PT values during external rotation.

This study has some limitations. First, this was a single-center, retrospective study, which may have introduced a selection bias. Additionally, this was an observational study. Therefore, causal relationships need to be validated through future multicenter, prospective studies. Second, the CSAs were manually delineated by only a single radiologist, which represents a significant limitation of this study. Therefore, comprehensive validation is necessary to establish both inter-reader and intra-reader reliabilities. In addition, the CSA was measured from a single slice in the Y-view. In cases of rotator cuff tears, this can result in an underestimation of the muscle CSA owing to retraction. Future studies could benefit from using AI-based automatic measurements to improve accuracy and consistency. Also, we anticipate that eCSA can be easily obtained from multiple slices with AI assistance. Third, we did not distinguish between the eCSA of the IST and TM. However, analyzing both muscles together may better reflect the Biodex Isokinetic Test results, as IST and TM function jointly as external rotators of the shoulder [35]. Unlike IST tendon tears, TM tendon tears were not analyzed separately, as isolated TM tendon tears are very rare [36]. Consequently, the analysis of this aspect was not significant. Moreover, no specific details regarding TM tendon tears were documented in the surgical records. Finally, this study did not include a healthy normal population, which limits the ability to establish normal reference values for eCSA. Furthermore, due to the cross-sectional design and the absence of long-term postoperative follow-up data, we were unable to evaluate the relationship between eCSA and functional recovery over time. Therefore, future studies should aim to include a healthy control group to define normal reference ranges for eCSA, enhancing its clinical applicability. Additionally, prospective, long-term follow-up studies are needed to clarify the relationship between changes in eCSA and postoperative functional outcomes, thereby validating eCSA as a prognostic indicator for rotator cuff muscle function recovery.

## CONCLUSION

This observational study demonstrated that the eCSA of the rotator cuff muscles serves as a valuable quantitative imaging marker for assessing muscle function. FL60TQ30, ER60PT, and EX60PT were identified as associational Biodex indices that can be used to independently evaluate the functions of SSc, ISTM, and SST. The present study demonstrated that the eCSA of the rotator cuff muscle can be utilized as a quantitative imaging marker for shoulder movement, particularly in the case of patients unable to participate in Isokinetic Test before surgery.

## Figures and Tables

**Fig. (3):**
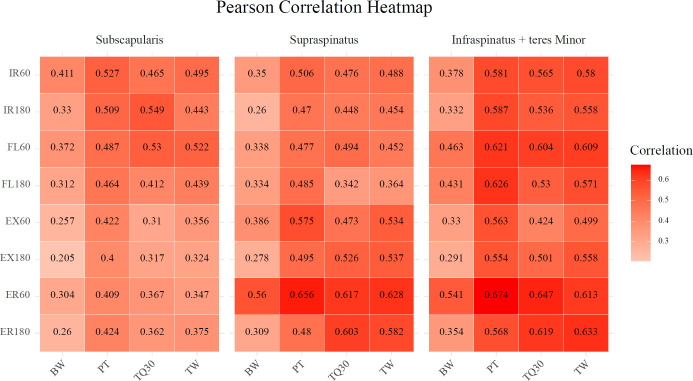
Pearson correlation matrix heat map.

**Table 1:** Baseline characteristics of the patients according to sex.

**Characteristics**	**Female** **(n = 45)**	**Male** **(n = 42)**	***p*-value**	**Total** **(n=87)**
Age (years)	63.6 ± 6.01	58.6 ± 9.44	0.005*	61.1 ± 8.20
BMI (kg/m^2^)	25.0 ± 3.51	25.5 ± 3.08	0.512	25.2 ± 3.30
Effective cross-sectional area (eCSA)				
SST_eCSA	304.3 ± 96.38	461.4 ± 148.71	<0.001*	380.1 ± 146.72
SSc_eCSA	1068.1 ± 274.36	1811.9 ± 452.97	<0.001*	1427.2 ± 525.43
ISTM_eCSA	880.1 ± 194.19	1398.4 ± 254.64	<0.001*	1130.3 ± 343.63
Biodex isokinetic test results				
FL60/PT	14.6 ± 6.26	26.9 ± 10.48	<0.001*	20.5 ± 10.54
FL60/BW	24.2 ± 10.53	37.0 ± 13.56	<0.001*	30.4 ± 13.61
FL60/TQ30	6.6 ± 6.13	17.1 ± 11.84	<0.001*	11.7 ± 10.69
FL60/TW	63.4 ± 48.30	156.1 ± 87.73	<0.001*	108.1 ± 83.88
EX60/PT	33.5 ± 10.05	54.3 ± 15.31	<0.001*	43.6 ± 16.53
EX60/BW	55.8 ± 16.21	75.1 ± 22.40	<0.001*	65.1 ± 21.61
EX60/TQ30	28.0 ± 10.91	45.2 ± 17.04	<0.001*	36.3 ± 16.57
EX60/TW	230.5 ± 105.20	377.7 ± 135.83	<0.001*	301.6 ± 141.17
FL180/PT	17.4 ± 5.99	31.1 ± 9.53	<0.001*	24.0 ± 10.45
FL180/BW	28.9 ± 10.99	42.6 ± 11.98	<0.001*	35.5 ± 13.34
FL180/TQ30	4.6 ± 3.89	11.3 ± 9.87	<0.001*	7.8 ± 8.10
FL180/TW	138.9 ± 90.47	321.9 ± 198.82	<0.001*	227.3 ± 177.47
EX180/PT	35.2 ± 12.47	52.7 ± 14.42	<0.001*	43.6 ± 15.99
EX180/BW	59.0 ± 20.65	73.0 ± 21.29	0.003*	65.7 ± 21.99
EX180/TQ30	17.2 ± 10.37	34.2 ± 18.99	<0.001*	25.4 ± 17.32
EX180/TW	493.2 ± 247.19	876.9 ± 404.86	<0.001*	678.5 ± 382.88
ER60/PT	7.4 ± 2.15	14.0 ± 5.48	<0.001*	10.6 ± 5.26
ER60/BW	12.3 ± 3.63	19.2 ± 6.73	<0.001*	15.6 ± 6.35
ER60/TQ30	4.6 ± 2.70	10.8 ± 6.33	<0.001*	7.6 ± 5.70
ER60/TW	31.2 ± 19.06	67.0 ± 36.71	<0.001*	48.5 ± 33.96
IR60/PT	17.6 ± 4.08	29.0 ± 9.87	<0.001*	23.1 ± 9.35
IR60/BW	29.5 ± 7.26	39.9 ± 12.17	<0.001*	34.5 ± 11.17
IR60/TQ30	14.3 ± 5.10	26.2 ± 11.19	<0.001*	20.1 ± 10.43
IR60/TW	98.3 ± 37.00	170.6 ± 66.31	<0.001*	133.2 ± 64.16
ER180/PT	9.0 ± 2.55	15.3 ± 5.00	<0.001*	12.0 ± 5.01
ER180/BW	15.1 ± 4.38	21.1 ± 6.86	<0.001*	18.0 ± 6.43
ER180/TQ30	3.0 ± 1.86	7.6 ± 4.65	<0.001*	5.2 ± 4.18
ER180/TW	65.8 ± 32.23	145.1 ± 70.83	<0.001*	104.1 ± 67.16
IR180/PT	19.6 ± 5.15	30.3 ± 8.69	<0.001*	24.8 ± 8.85
IR180/BW	33.0 ± 9.27	41.9 ± 11.55	<0.001*	37.3 ± 11.29
IR180/TQ30	10.9 ± 5.40	21.0 ± 8.56	<0.001*	15.8 ± 8.69
IR180/TW	237.3 ± 94.90	425.0 ± 165.42	<0.001*	327.9 ± 162.92
Hypertension, n (%)			0.474	
No	27 (60.0)	22 (52.4)		49 (56.3)
Yes	18 (40.0)	20 (47.6)		38 (43.7)
Diabetes mellitus, n (%)			1.000	
No	40 (88.9)	38 (90.5)		78 (89.7)
Yes	5 (11.1)	4 (9.5)		9 (10.3)
Alcohol consumption, n (%)			<0.001*	
None	43 (95.6)	18 (42.9)		61 (70.1)
Yes	2 (4.4)	24 (57.1)		26 (29.9)
Smoking status, n (%)			<0.001*	
None	42 (93.3)	18 (42.9)		60 (69.0)
Former smoker	3 (6.7)	11 (26.2)		14 (16.1)
Current smoker	0 (0.0)	13 (31.0)		13 (14.9)
SST tear, n (%)			0.714	
Normal	3 (6.7)	5 (11.9)		8 (9.2)
Partial-thickness tear	12 (26.7)	10 (23.8)		22 (25.3)
Full-thickness tear	30 (66.7)	27 (64.3)		57 (65.5)
IST tear, n (%)			0.372	
Normal	27 (60.0)	19 (45.2)		46 (52.9)
Partial thickness tear	3 (6.7)	5 (11.9)		8 (9.2)
Full-thickness tear	15 (33.3)	18 (42.9)		33 (37.9)
SSc tear^†^, n (%)			0.237	
0	17 (37.8)	12 (28.6)		29 (33.3)
1	17 (37.8)	14 (33.3)		31 (35.6)
2a	7 (15.6)	5 (11.9)		12 (13.8)
2b	2 (4.4)	5 (11.9)		7 (8.0)
3	0 (0.0)	4 (9.5)		4 (4.6)
4	2 (4.4)	1 (2.4)		3 (3.4)
5	0 (0.0)	1 (2.4)		1 (1.1)
LHBT tear, n (%)			0.290	
Normal	24 (53.3)	16 (38.1)		40 (46.0)
Partial tear	20 (44.4)	23 (54.8)		43 (49.4)
Total rupture	1 (2.2)	3 (7.1)		4 (4.6)

**Abbreviations:** SST, supraspinatus; SSc, subscapularis; ISTM, infraspinatus and teres minor; IST, infraspinatus; LHBT, long head of the biceps tendon; PT, peak torque; BW, peak torque/body weight; TQ30, torque at 30°; TW, total work. The Yoo and Rhee classification was used for subscapularis tear grading. * *p* < 0.05. Continuous values are presented as the mean ± standard deviation.

**Table 2:** Univariate regression analyses and GLM of the eCSA of SSc and Biodex results with high correlation coefficients.

-	**Biodex Test**	**IR180TQ30**				**FL60TQ30**			
		**Univariate Regression Analysis**		**GLM**		**Univariate Regression Analysis**		**GLM**	
**Variables**		**Odds Ratio (95% CI)**	***p*-value**	**β-coefficient**	***p*-value**	**Odds Ratio (95% CI)**	***p*-value**	**β-coefficient**	***p*-value**
eCSA of SSc	-	-	-	0.00 (0.00, 0.01)	0.059	-	-	0.01 (0.00, 0.01)	0.021*
Age (years)	-	-0.32 (-0.52, -0.10)	0.004*	-0.13 (-0.33, 0.06)	0.2	-0.38 (-0.65, -0.11)	0.007*	-0.10 (-0.36, 0.15)	0.4
Sex	Female	-	-	-	-	-	-	-	-
-	Male	10 (7.1, 13)	<0.001*	6.1 (1.4, 11)	0.011*	11 (6.6, 15)	<0.001*	5.2 (-0.85, 11)	0.091
BMI (kg/m^2^)	-	-0.08 (-0.64, 0.49)	0.8	-	-	0.78 (0.10, 1.5)	0.026*	0.57 (-0.04, 1.2)	0.065
Hypertension	No	-	-	-	-	-	-	-	-
-	Yes	2.4 (-1.3, 6.2)	0.2	-	-	1.8 (-2.8, 6.4)	0.5	-	-
Diabetes mellitus	No	-	-	-	-	-	-	-	-
-	Yes	3.0 (-3.1, 9.1)	0.3	-	-	-0.88 (-8.4, 6.6)	0.8	-	-
Alcohol consumption	None	-	-	-	-	-	-	-	-
-	Yes	6.6 (2.8, 10)	<0.001*	0.34 (-3.8, 4.4)	0.9	6.6 (1.8, 11)	0.008*	0.14 (-5.3, 5.6)	>0.9
Smoking status	None	-	-	-	-	-	-	-	-
-	Former smoker	8.1 (3.3, 13)	0.001*	3.0 (-1.6, 7.5)	0.2	3.2 (-2.8, 9.2)	0.3	-2.2 (-8.1, 3.7)	0.5
-	Current smoker	7.0 (2.1, 12)	0.006*	-1.7 (-7.0, 3.7)	0.5	9.9 (3.7, 16)	0.002*	0.06 (-6.8, 6.9)	>0.9
SST tear	Normal	-	-	-	-	-	-	-	-
-	Partial-thickness tear	-3.0 (-10, 4.2)	0.4	-	-	2.3 (-6.3, 11)	0.6	-	-
-	Full-thickness tear	-4.2 (-11, 2.4)	0.2	-	-	-3.6 (-11, 4.3)	0.4	-	-
IST tear	Normal	-	-	-	-	-	-	-	-
-	Partial-thickness tear	3.9 (-2.8, 10)	0.3	-	-	2.3 (-5.9, 10)	0.6	-	-
-	Full-thickness tear	-0.64 (-4.6, 3.3)	0.7	-	-	-1.6 (-6.4, 3.3)	0.5	-	-
SSc tear	0	-	-	-	-	-	-	-	-
-	1	3.9 (-0.37, 8.2)	0.073	-	-	0.60 (-4.9, 6.1)	0.8	-	-
-	2a	-4.9 (-11, 0.75)	0.088	-	-	-4.9 (-12, 2.4)	0.2	-	-
-	2b~5	2.4 (-2.8, 7.7)	0.4	-	-	-1.5 (-8.3, 5.2)	0.7	-	-
LHBT tear	Normal	-	-	-	-	-	-	-	-
-	Partial tear	-2.0 (-5.8, 1.9)	0.3	-	-	-0.84 (-5.6, 3.9)	0.7	-	-
-	Complete tear	-3.3 (-12, 5.8)	0.5	-	-	-2.5 (-14, 8.8)	0.7	-	-

**Abbreviations:** SST, supraspinatus; SSc, subscapularis; ISTM, infraspinatus and teres minor; IST, infraspinatus; LHBT, long head of the biceps tendon; TQ30, torque at 30°. The Yoo and Rhee classification was used for subscapularis tear grading. * p < 0.05.

**Table 3:** Univariate regression analyses and GLM of the eCSA of SST and Biodex results with high correlation coefficients.

-	**Biodex Test**	**ER60PT**				**EX60PT**			
		**Univariate Regression Analysis**		**GLM**		**Univariate Regression Analysis**		**GLM**	
Variables		Odds Ratio (95% CI)	*p*-value	β-coefficient	*p*-value	Odds Ratio (95% CI)	*p*-value	β-coefficient	*p*-value
eCSA of SST	-	-	-	0.01 (0.01, 0.02)	0.001	-	-	0.03 (0.00, 0.06)	0.023
Age (years)	-	-0.29 (-0.42, -0.17)	<0.001	-0.11 (-0.22, 0.00)	0.046	-0.62 (-1.0, -0.20)	0.004	-0.16 (-0.51, 0.19)	0.4
Sex	Female	-	-	-	-	-	-	-	-
-	Male	6.6 (4.9, 8.4)	<0.001	3.1 (0.47, 5.7)	0.021	21 (15, 26)	<0.001	17 (9.7, 25)	<0.001
BMI (kg/m^2^)	-	0.25 (-0.09, 0.59)	0.14	-	-	0.69 (-0.38, 1.8)	0.2	-	-
Hypertension	No	-	-	-	-	-	-	-	-
-	Yes	0.82 (-1.5, 3.1)	0.5	-	-		>0.9	-	-
Diabetes mellitus	No	-	-	-	-	-	-	-	-
-	Yes	0.18 (-3.5, 3.9)	>0.9	-	-	4.5 (-7.1, 16)	0.4	-	-
Alcohol consumption	None	-	-	-	-	-	-	-	-
-	Yes	2.7 (0.31, 5.1)	0.028	-1.0 (-3.2, 1.2)	0.4	-0.20 (-7.3, 6.9)	0.089	-	-
Smoking status	None	-	-	-	-	-	-	-	-
-	Former smoker	3.9 (0.93, 6.8)	0.011	1.4 (-0.84, 3.7)	0.2	11 (1.2, 20)	0.028	0.36 (-7.1, 7.8)	>0.9
-	Current smoker	4.0 (0.97, 7.0)	0.010	1.3 (-1.4, 4.0)	0.4	5.2 (-4.6, 15)	0.3	-9.8 (-18, -1.1)	0.027
SST tear	Normal	-	-	-	-	-	-	-	-
-	Partial-thickness tear	-3.7 (-7.9, 0.46)	0.080	-0.61 (-3.7, 2.5)	0.7	-16 (-30, -3.2)	0.015	-11 (-21, -1.5)	0.024
-	Full-thickness tear	-5.1 (-8.9, -1.3)	0.010	-0.23 (-3.2, 2.7)	0.9	-17 (-29, -4.6)	0.007	-5.6 (-15, 4.2)	0.3
IST tear	Normal	-	-	-	-	-	-	-	-
-	Partial-thickness tear	1.2 (-2.9, 5.2)	0.6	-	-	-0.68 (-13, 12)	>0.9	-	-
-	Full-thickness tear	-0.97 (-3.4, 1.4)	0.4	-	-	-0.73 (-8.3, 6.9)	0.8	-	-
SSc tear	0	-	-	-	-	-	-	-	-
-	1	1.2 (-1.4, 3.8)	0.4	0.92 (-0.91, 2.8)	0.3	3.0 (-5.2, 11)	0.5	2.4 (-3.5, 8.3)	0.4
-	2a	-1.4 (-4.8, 2.1)	0.4	-0.79 (-3.3, 1.7)	0.5	-8.6 (-19, 2.2)	0.12	-8.2 (-16, -0.18)	0.045
-	2b~5	4.5 (1.4, 7.7)	0.006	3.6 (1.1, 5.9)	0.005	11 (1.1, 21)	0.030	2.0 (-5.9, 9.9)	0.6
LHBT tear	Normal	-	-	-	-	-	-	-	-
-	Partial tear	-0.56 (-2.9, 1.7)	0.6	-	-	-4.1 (-11, 3.0)	0.3	-	-
-	Complete tear	3.7 (-1.8, 9.1)	0.2	-	-	11 (-5.7, 28)	0.2	-	-

**Abbreviations:** SST, supraspinatus; SSc, subscapularis; ISTM, infraspinatus and teres minor; IST, infraspinatus; LHBT, long head of the biceps tendon; PT, peak torque. The Yoo and Rhee classification was used for subscapularis tear grading. * *p* < 0.05.

**Table 4:** Univariate regression analyses and GLM of the eCSA of ISTM and Biodex results with high correlation coefficients.

-	**Biodex Test**	**ER60PT**				**FL180PT**			
		**Univariate Regression Analysis**		**GLM**		**Univariate Regression Analysis**		**GLM**	
**Variables**		**Odds Ratio (95% CI)**	***p*-value**	**β-coefficient**	***p*-value**	**Odds Ratio (95% CI)**	***p*-value**	**β-coefficient**	***p*-value**
eCSA of ISTM	-	-	-	0.01 (0.00, 0.01)	<0.001	-	-	0.01 (0.00, 0.02)	0.032
Age (years)	-	-0.29 (-0.42, -0.17)	<0.001	-0.15 (-0.25, - 0.05)	0.004	-0.42 (-0.68, -0.16)	0.002	-0.1 (-0.31, 0.11)	0.3
Sex	Female	-	-	-	-	-	-	-	-
-	Male	6.6 (4.9, 8.4)	<0.001	2.7 (0.01, 5.4)	0.049	14 (10, 17)	<0.001	11 (4.6, 16)	0.001
BMI (kg/m^2^)	-	0.25 (-0.09, 0.59)	0.14	-	-	0.68 (0.01, 1.3)	0.047	0.4 (-0.11, 0.90)	0.142
Hypertension	No	-	-	-	-	-	-	-	-
-	Yes	0.82 (-1.5, 3.1)	0.5	-	-	2.3 (-2.2, 6.8)	0.3	-	-
Diabetes mellitus	No	-	-	-	-	-	-	-	-
-	Yes	0.18 (-3.5, 3.9)	>0.9	-	-	-0.40 (-7.8, 7.0)	>0.9	-	-
Alcohol consumption	None	-	-	-	-	-	-	-	-
-	Yes	2.7 (0.31, 5.1)	0.028	-1.8 (-.3.9, 0.32)	0.1	7.2 (2.5, 12)	0.003	-0.05 (-4.7, 4.6)	>0.9
Smoking status	None	-	-	-	-	-	-	-	-
-	Former smoker	3.9 (0.93, 6.8)	0.011	1.0 (-1.2, 3.3)	0.4	1.8 (-4.2, 7.8)	0.5	-4.5 (-9.3, 0.39)	0.071
-	Current smoker	4.0 (0.97, 7.0)	0.010	0.10 (-2.5, 2.7)	>0.9	8.6 (2.5, 15)	0.007	-2.6 (-8.2, 3.0)	0.4
SST tear	Normal	-	-	-	-	-	-	-	-
-	Partial-thickness tear	-3.7 (-7.9, 0.46)	0.080	-0.12 (-.3.2, 3.0)	>0.9	-1.8 (-10, 6.8)	0.7	-	-
-	Full-thickness tear	-5.1 (-8.9, -1.3)	0.010	-1.3 (-4.1, 1.5)	0.4	-4.1 (-12, 3.7)	0.3	-	-
IST tear	Normal	-	-	-	-	-	-	-	-
-	Partial-thickness tear	1.2 (-2.9, 5.2)	0.6	-	-	3.9 (-4.1, 12)	0.3	-	-
-	Full-thickness tear	-0.97 (-3.4, 1.4)	0.4	-	-	-0.08 (-4.9, 4.7)	>0.9	-	-
SSc tear	0	-	-	-	-	-	-	-	-
-	1	1.2 (-1.4, 3.8)	0.4	1.2 (-0.63, 3.0)	0.2	-1.4 (-6.7, 3.9)	0.6	-	-
-	2a	-1.4 (-4.8, 2.1)	0.4	-0.82 (-3.3, 1.7)	0.5	-6.5 (-14, 0.56]	0.071	-	-
-	2b–5	4.5 (1.4, 7.7)	0.006	3.3 (0.93, 5.6)	0.007	2.5 (-4.0, 9.0)	0.4	-	-
LHBT tear	Normal	-	-	-	-	-	-	-	-
-	Partial tear	-0.56 (-2.9, 1.7)	0.6	-	-	-1.1 (-5.7, 3.4)	0.6	-	-
-	Complete tear	3.7 (-1.8, 9.1)	0.2	-	-	6.8 (-4.1, 18)	0.2	-	-

**Abbreviations:** SST, supraspinatus; SSc, subscapularis; ISTM, infraspinatus and teres minor; IST, infraspinatus; LHBT, long head of the biceps tendon; PT, peak torque. The Yoo and Rhee classification was used for subscapularis tear grading. * *p* < 0.05.

## Data Availability

The datasets generated or analyzed during the study are available from the corresponding author [C.H.K] on reasonable request.

## LIST OF ABBREVIATIONS

CSA: Cross-Sectional Area
eCSA: Effective Cross-Sectional Area
SSc: Subscapularis
SST: Supraspinatus
IST: Infraspinatus
TM: Teres Minor
ISTM: Infraspinatus and Teres Minor
FL: Flexion
EX: Extension
IR: Internal Rotation
ER: External Rotation
PT: Peak Torque
BW: Peak Torque/Body Weight
TQ30: Torque at 30˚
TW: Total Work
BMI: Body Mass Index
LHBT: Long Head of Biceps Tendon
GLM: General Linear Model
AIC: Akaike Information Criterion
CT: Computed Tomography

## STANDARDS OF REPORTING

STROBE guidelines were followed.
